# Effect of Hydrocolloids on Penetration Tests, Sensory Evaluation, and Syneresis of Milk Pudding

**DOI:** 10.3390/polym17030300

**Published:** 2025-01-23

**Authors:** Hong-Ting Victor Lin, Jenn-Shou Tsai, Hsiao-Hui Liao, Wen-Chieh Sung

**Affiliations:** 1 Department of Food Science, National Taiwan Ocean University, Keelung 20231, Taiwan; hl358@mail.ntou.edu.tw (H.-T.V.L.); tsaijs@mail.ntou.edu.tw (J.-S.T.); sammi_liao@weichuan.com.tw (H.-H.L.); 2 Center of Excellence for the Oceans, National Taiwan Ocean University, Keelung 20231, Taiwan

**Keywords:** milk pudding, carrageenans, gellan gum, sensory evaluation, penetration test, syneresis, agar

## Abstract

This study evaluated how added gums, starch amounts, and sucrose levels affect the texture, sensory acceptability, and syneresis of milk puddings. The puddings were prepared with four ingredients, namely 0.3% polysaccharide (κ,ι-carrageenan, gellan gum, gelatin, or agar), 2.5–7.5% sucrose, 1–5% modified waxy corn starch, and whole milk. The physical and sensory properties were assessed through measurements of gel strength, breaking point, breaking force, rigidity, and hedonic testing. Results show that syneresis increased in all milk puddings during two weeks of refrigerated storage. Among the five polysaccharides, agar and κ-carrageenan showed the most significant effect on gel rigidity and strength, while gellan gum and ι-carrageenan were more effective at preventing syneresis compared to three commercial milk pudding products after 14 days of storage. As modified corn starch concentration increased (1 to 5%), gel strength, breaking force, and rigidity decreased. Lower modified waxy corn starch concentrations (5% to 1%) led to reduced syneresis when stored at 4 °C for 7 and 14 days. Sucrose significantly increased gel strength and breaking force in puddings containing κ-carrageenan, gellan gum, and agar. Gellan gum improved overall pudding acceptability. Based on sensory and syneresis data, the most acceptable milk pudding formulation contained 5% sucrose, 0.3% gellan gum, and 1% modified waxy corn starch.

## 1. Introduction

Milk pudding, a widely consumed dairy product globally, incorporates milk, sucrose, and hydrocolloids in its formulation, appealing to diverse consumer demographics, including children, students, and elderly individuals. The primary hydrocolloids utilized in dairy dessert production are modified starch and carrageenans [1,2]. As a carbohydrate component, starch comprises linear amylose and branched amylopectin structures. Incorporating modified starch enhances the product’s textural qualities, while carrageenan contributes specific structural characteristics. κ-carrageenan provides firmness and brittleness, whereas ι-carrageenan yields elasticity and softness [3]. The combination of hydrocolloids represents an effective approach to enhance both texture and starch functionality in gelled food products, surpassing the performance of individual components [4]. During processing, κ-carrageenan and ι-carrageenan form thermoreversible hydrogels through interaction swith gel-promoting cations during thermal treatment and cooling [5]. The presence of starch and milk proteins significantly influences carrageenan gel formation. Specifically, milk proteins enhance gel strength, while increased concentrations of both carrageenan and starch reduce syneresis [6]. Starch–gum systems’ characteristics depend on multiple variables, including gum and starch types, concentrations, and processing parameters. The interactions between milk proteins and carrageenan and their subsequent effects on gel structure and texture have been extensively studied [5,6,7,8,9].

Research examining gum–starch complexes demonstrates enhanced viscosity compared to individual components [10]. Texture represents a crucial quality parameter in milk pudding production, with stabilizers serving as thickening or gelling agents—including agar, gelatin, and various hydrocolloids—contributing to optimal texture and stability [11,12]. In dairy dessert formulations, sugar exhibits an inhibitory effect on starch granule swelling through water competition, thereby affecting gelatinization temperature [13]. However, the mechanism of sugar’s influence on starch–gum interactions requires further investigation. Research by Lethuant et al. [14] examined texture–sweetness relationships in dairy dessert formulations with varying carrageenan compositions and sucrose levels. Their findings indicated a direct correlation between sucrose content and sweetness across different carrageenan types (κ-, ι-, λ-carrageenans). The sensory perception of dairy desserts is inherently linked to their structural composition, which can be optimized through thickening or gelling agent incorporation [14].

Syneresis, characterized by the expulsion of entrapped water from viscoelastic gel networks, varies with hydrocolloid type and molecular dimensions. While hydrocolloids enhance mouthfeel and reduce syneresis [15,16] through water retention within the network structure, certain polysaccharides interact with proteins in gel systems to modify hydration characteristics [17]. Carrageenans, derived from red algae as sulfated polysaccharides, primarily consist of D-galactans and 3,6-anhydride galactans. When combined with potassium chloride, κ-carrageenan forms relatively brittle and firm gels that are prone to syneresis [18,19]. This syneresis effect, manifesting as free water release, has been documented in dynamic measurements [20]. The addition of specific gums, such as konjac glucomannan and locust bean gum, effectively minimizes syneresis in carrageenan systems [21].

Syneresis extent correlates with starch retrogradation tendency [22]. Starch retrogradation involves the recrystallization and aggregation of gelatinized starch molecules. The increased firmness observed in starch-based products during storage primarily results from amylopectin retrogradation. Gum–starch molecular interactions can effectively inhibit starch retrogradation. Certain gums effectively control both retrogradation and syneresis in milk pudding formulations. The product typically consists of two distinct phases, an aqueous continuous phase containing modified maize starch and gum and a dispersed phase comprising milk fat and sugar stabilized by proteins. Hydrocolloids mitigate starch retrogradation by modifying starch–water interactions. Current research on hydrocolloid and sucrose effects regarding penetration testing, texture sensory analysis, and syneresis in milk pudding remains limited. Excessive drainage and sweetness in milk pudding products generally receive negative consumer feedback and raise health concerns. This study aims to examine the interactions between various hydrocolloids (including carrageenans, gellan gum, gelatin, and agar) with modified corn starch and sucrose in whole milk formulations, analyzing penetration test parameters, syneresis characteristics, and sensory evaluations. The research objective focuses on improving syneresis control and texture optimization through varied hydrocolloid incorporation, sucrose levels, and modified corn starch concentrations, ultimately developing enhanced formulations and improving existing commercial products during refrigerated storage at 4 °C over 14 days.

## 2. Materials and Methods

### 2.1. Raw Materials

The primary milk source utilized was obtained from Wei Chuan Corp (Lujhu, Kaohsiung, Taiwan). The stabilizing agents and thickeners were supplied by Gemfont Corporation (Taipei, Taiwan), including modified waxy corn starch (CH-20), κ-carrageenan (WR-78G), ι-carrageenan (RMD-102), gellan gum (LT-100), gelatin (L200), and agar. The modified waxy corn starch (CLEARAM^®^ CH 20, Roquette, Lestrem, France), processed through drum drying, served as a thickening agent (E1422) commonly employed in various food applications. The κ-carrageenan (GENUGEL^®^ WR-78G, Cpkelco, Chicago, IL, USA) consisted of purified kappa-structured carrageenan extracted from select red algae species. The ι-carrageenan (GENUGEL^®^ carrageen type RMD-102, Cpkelco, Chicago, IL, USA) was similarly derived from red algae. The high-acyl gellan gum (KELCOGEL^®^ LT-100, Cpkelco, Chicago, IL, USA) was produced through fermentation. The gelatin powder (VIDOGUM L200, UNIPERKIN, Thiurgau, Switzerland) was extracted from pork skin, exhibiting a gel strength of 200 bloom and viscosity ranging from 700 to 2300 mPa.s. Agar C powder was obtained from *Gracilaria* seaweed harvested from various coastal regions. Sucrose was sourced from Taiwan Sugar Corporation (Siaogang, Kaohsiung, Taiwan).

### 2.2. Milk Pudding Preparation

The milk pudding formulation consisted of 0.3% (*w*/*w*) hydrocolloids (kappa-carrageenan, lambda-carrageenan, gellan gum, gelatin, or agar), modified waxy corn starch (1%, 3%, or 5% (*w*/*w*)), sucrose (2.5%, 5%, or 7.5% (*w*/*w*)), and whole milk, combined to achieve a total mass of 1000 g. The mixture underwent thorough homogenization followed by sterilization in an autoclave (HL-380 Fully Automatic High-Pressure Sterilizer, Hanlein, Taiwan) at 100 °C for 20 min, maintaining chamber pressure below 1.2 Kg/cm^2^. The prepared solutions were distributed into standardized glass beakers (20 mL, Φ 21 mm, H33 mm) and polypropylene cups (100 mL, Φ 70 mm on top, Φ 40 mm at bottom, H54 mm). Following equilibration to room temperature (25–27 °C), samples were refrigerated at 4 °C for subsequent analysis at 7 and 14 days [4].

### 2.3. Analysis of Textural Characteristics

Textural properties were evaluated using a Model TA-XT2 texture analyzer (Stable Micro System, Haslemere, UK). Six samples per treatment underwent analysis for breaking point (B.P.) and breaking force (B.F.), utilizing a 5 mm diameter spherical plunger at 100 mm/min [6]. The following equations were employed to determine gel strength (G.S.) and rigidity (R.) [23]:

Gel strength (g/mm) = breaking force (g) × breaking point (mm)

Rigidity (g/mm) = breaking force (g)/breaking point (mm)

All measurements were conducted in duplicate at ambient temperature (25–27 °C).

### 2.4. Measurement % Syneresis

Following the methodology of Charoenrein et al. [24], syneresis was evaluated in milk pudding samples. Products were stored with coverage at 4 °C for 7 and 14 days. The initial sample weight (w1) was recorded prior to storage. After the storage period, the expelled liquid was carefully decanted for 2 min, and the remaining sample was weighed (w2). Syneresis was calculated as (w1 − w2)/w1 and expressed as a percentage.

### 2.5. Sensory Evaluation

The sensory panel comprised 30 participants (15 females, 15 males) from the Department of Food Science, National Taiwan Ocean University, including students, staff, and faculty members. All participants had previous sensory evaluation experience and were familiar with commercial milk pudding products. Prior to evaluation, panelists received training on identifying key attributes of commercial milk pudding, utilizing hedonic scales and defining intensity parameters for color, flavor, sweetness, springiness, and overall acceptability [25]. Participants were specifically trained to assess sweetness and springiness characteristics in formulated dairy desserts and subsequently evaluated three commercial milk pudding products.

Samples were presented under controlled conditions, identified by three-digit codes, and they were maintained at refrigeration temperature. Commercial products were evaluated using a 9-point hedonic scale (1 = dislike extremely; 5 = neither like nor dislike; 9 = like extremely) [26]. Evaluations were conducted in a designated testing area, with water provided for palate cleansing between samples. Data represented the mean of thirty evaluations. The same panel evaluated both commercial and test samples, with 4–5 samples assessed per session. Evaluations utilized an intensity score sheet, comparing samples against a commercial milk pudding (MA) control. Assessment proceeded systematically; first, an analysis of color and flavor intensity was conducted, followed by a sweetness and springiness evaluation using a separate sample portion, concluding with an overall acceptability assessment.

### 2.6. Statistical Analysis

Statistical analysis employed ANOVA methodology. Significant differences among means were determined using Duncan’s multiple range test at the 5% significance level (*p* < 0.05). All analyses were performed using SPSS 12.0 (SPSS Inc., Chicago, IL, USA).

## 3. Results

### 3.1. Analysis of Textural Characteristics and % Syneresis

**Figure 1:**
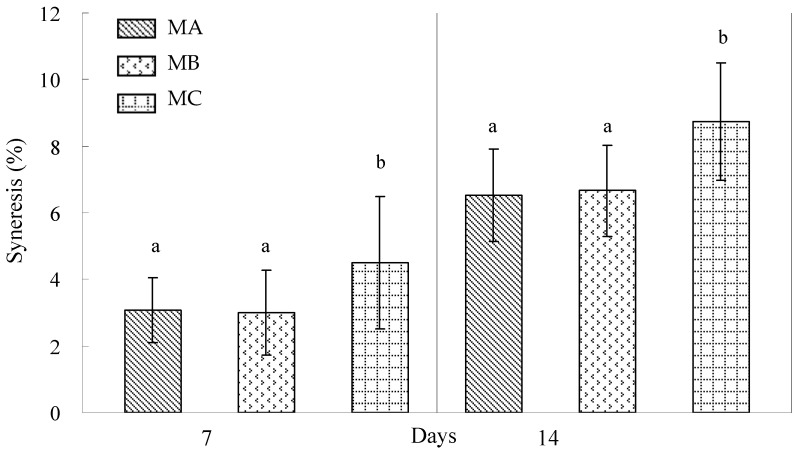
Syneresis of different commercial milk pudding products stored for 7 days and 14 days at 4 °C. Each data point was the mean of 6 determinations. Means with a different letter for the same storage day are significantly different (*p* < 0.05).

An analysis of penetration tests conducted on three commercial milk puddings (Table 1) indicated that commercial milk pudding MC exhibited the highest values across all measured parameters (*p* < 0.05). The penetration test revealed that milk pudding MB demonstrated significantly lower values in breaking force, gel strength, and rigidity (*p* < 0.05) compared to the other two products (Table 1). The comparative analysis of syneresis measurements for the three commercial milk pudding products is presented in Figure 1.

Table 2 presents the penetration test analysis of milk pudding samples containing five polysaccharides, evaluated at concentrations of 0.3% and 1% modified corn starch, stored at 4 °C for 7 days. The analysis revealed that milk pudding formulated with 0.3% agar exhibited superior breaking force, gel strength, and rigidity compared to other formulations (*p* < 0.05).

**Table 2:** Effect of modified corn starch (1%) and different polysaccharides (0.3%) on textural characteristics ^1^ of milk pudding penetration test (mean ± SD, n = 6).

Type of Polysaccharide	Breaking Force (B.F.)	Breaking Point (B.P.)	Gel Strength (G.S.)	Rigidity (R.)
	(g)	(mm)	(g × mm)	(g/mm)
κ-carrageenan	22.7 ± 0.3 ^d^	5.41 ± 0.14 ^c^	123 ± 4 ^d^	4.20 ± 0.10 ^d^
ι-carrageenan	11.7 ± 0.3 ^a^	6.34 ± 0.13 ^e^	74 ± 3 ^a^	1.84 ± 0.02 ^a^
Gellan gum	18.8 ± 0.5 ^c^	5.23 ± 0.10 ^a^	98 ± 3 ^c^	3.59 ± 0.12 ^c^
Gelatin	14.6 ± 0.5 ^b^	6.25 ± 0.14 ^d^	91 ± 3 ^b^	2.34 ± 0.08 ^b^
Agar	26.6 ± 0.4 ^e^	5.37 ± 0.03 ^b^	143 ± 2 ^e^	4.95 ± 0.06 ^e^

^1^ Gel strength (G.S.) = (Breaking force × Breaking point); Rigidity = (Breaking force/Breaking point). Data are expressed as mean (n = 6). Different superscripts in the same column indicate significant differences. Figure 2 illustrates the impact of polysaccharides on % syneresis in milk pudding during refrigerated storage at 4 °C over 7 and 14 days. These findings align with previous research examining binary hydrocolloid gel systems (0.5 wt%) comprising agar, gellan gum, and gelatin under similar storage conditions [4]. The analysis demonstrates that milk pudding formulated with gellan gum exhibited significantly reduced syneresis levels throughout the storage period. Notably, milk pudding containing 0.3% κ-carrageenan demonstrated minimal syneresis progression, increasing from 5.83% at day 7 to 7.17% at day 14. The result shows that both ι-carrageenan and gellan gum serve as effective agents in minimizing syneresis in milk pudding formulations.

**Figure 2:**
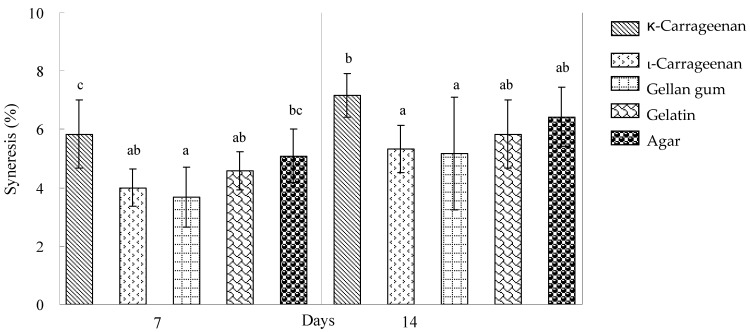
Effect of different polysaccharides on syneresis of milk pudding stored for 7 days and 14 days at 4 °C. Each data point represents the mean of 6 determinations. Means with a different letter for the same storage day are significantly different (*p* < 0.05).

**Figure 3:**
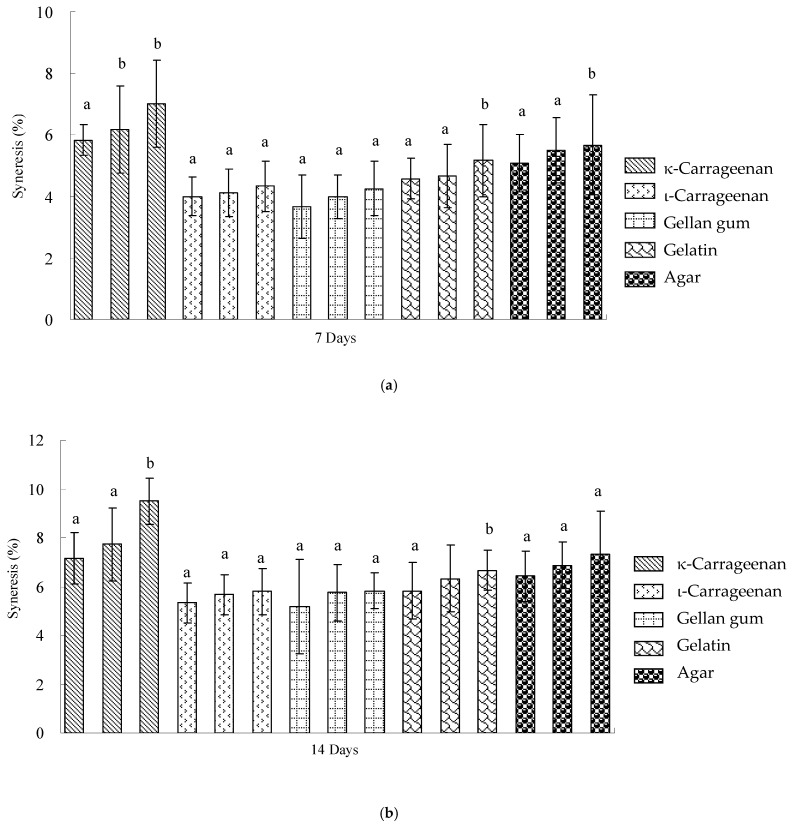
(**a**). Effect of various gums (0.3%) and different concentrations of modified corn starch (1, 3, and 5%) on the syneresis of milk pudding stored at 4 °C for 7 days. Each data point was the mean of 6 determinations. Means with a different letter in the same column are significantly different (*p* < 0.05). (**b**) The effect of various gums (0.3%) and different concentrations of modified corn starch (1, 3, and 5%) on the syneresis of milk pudding stored at 4 °C for 14 days. Each data point was the mean of 6 determinations. Means with a different letter in the same column are significantly different (*p* < 0.05). The addition of sucrose generally increased all parameter characteristics of milk pudding for penetration tests (Table 4). Sucrose significantly promoted gel strength and the breaking force of milk pudding containing the κ-carrageenan, gellan gum, and agar (*p* < 0.05; Table 4).

Table 3 demonstrates the effects of varying polysaccharide types and modified waxy corn starch concentrations on the penetration test parameters of milk pudding. As illustrated in Figure 3a,b, the reduction in modified waxy corn starch concentration correlates with decreased syneresis percentage, particularly evident at 1% starch concentration. The % syneresis observed in milk puddings containing 1% modified waxy corn starch and 0.3% of various gums (Figure 3a,b) exhibited comparable values to milk pudding gels containing 0.3% gum alone during the 7-day and 14-day refrigerated storage periods (Figure 2). Notably, the addition of gellan gum and ι-carrageenan to milk puddings resulted in negligible variations in syneresis across different modified corn starch concentrations throughout both storage durations.

### 3.2. Sensory Evaluation of Milk Pudding

Table 5 presents the sensory analysis results comparing commercial and 0.3% polysaccharide milk pudding products. Among the evaluated parameters, namely color, flavor, sweetness, springiness, and overall acceptability, milk puddings MB and MC demonstrated comparable mean scores, whereas MA exhibited superior ratings (*p* < 0.05).

The hedonic sensory evaluation data for milk puddings containing various polysaccharides are documented in Table 5. The analysis reveals that incorporating 5% sucrose with different polysaccharides maintained consistent sweetness and flavor profiles across samples (*p* > 0.05). Milk puddings containing 0.3% gellan gum and ι-carrageenan demonstrated enhanced color attributes compared to those with 0.3% κ-carrageenan, gelatin, and agar (*p* < 0.05). In terms of springiness, formulations with 0.3% gelatin, gellan gum, and ι-carrageenan exhibited superior characteristics relative to those containing 0.3% κ-carrageenan and agar (*p* < 0.05). The 0.3% gellan gum formulation achieved higher overall acceptability compared to variants containing 0.3% gelatin and ι-carrageenan (*p* > 0.05), as well as those with κ-carrageenan and agar (*p* < 0.05).

The assessment of milk puddings formulated with varying sucrose concentrations (2.5%, 5%, and 7.5%) involved 30 panelists evaluating four samples, including a commercial product (MA), using a nine-point hedonic scale. The analysis indicated comparable ratings for color, flavor, springiness, and overall acceptability between formulations containing 5% and 7.5% sucrose, with sweetness being the sole distinguishing factor (Table 6). Specifically, the formulation incorporating 0.3% gellan gum and 1% modified corn starch with 5% sucrose received higher sweetness ratings compared to its counterpart containing 7.5% sucrose (*p* < 0.05).

## 4. Discussion

### 4.1. Analysis of Textural Characteristics and % Syneresis

The analysis reveals that commercial milk pudding products MA and MB exhibited significantly lower syneresis levels (*p* < 0.05) compared to MC during refrigerated storage at both 7 and 14 days. The commercial milk puddings demonstrated syneresis values ranging from 3.00 to 4.50% after 7 days of storage, which subsequently increased substantially to 6.53–8.73% after 14 days. This represents an approximate 109% increase in syneresis values during extended refrigerated storage.

Research indicates that agar enhances breaking force, gel strength, and rigidity while providing a firm brittle texture in low concentrations (0.1–0.5%) when combined with modified corn starch gel [4]. At 0.5% concentration, agar gel demonstrated superior gel strength, rigidity, and breaking force compared to equivalent concentrations of gellan gum and gelatin in corn starch gel systems [4]. Milk pudding containing 0.3% ι-carrageenan exhibited the lowest breaking force, gel strength, and rigidity among the polysaccharide variants (*p* < 0.05). However, it maintained a higher breaking point relative to other formulations (*p* < 0.05). The incorporation of gellan gum resulted in reduced breaking points (*p* < 0.05). When combined with gelatin, gellan gum enhanced the breaking point, breaking force, and gel strength, though these improvements were less pronounced compared to agar–gelatin combinations at equivalent ratios [4]. Previous studies have demonstrated that agar or κ-carrageenan additions increase breaking force, gel strength, and rigidity while decreasing breaking point values [3]. Present findings confirm that agar and κ-carrageenan contribute to firm brittle textures, while ι-carrageenan reduces breaking force, gel strength, and rigidity while increasing the breaking point [3]. ι-carrageenan effectively imparts soft elastic characteristics to milk pudding formulations.

The measurement of % syneresis in milk pudding indicates starch’s capacity to prevent undesirable liquid separation during refrigeration. This phenomenon occurs due to increased molecular associations between amylose and amylopectin components, particularly through linear amylose retrogradation [27], resulting in water expulsion from the gel matrix [28]. The extent of water separation serves as an effective measure of starch retrogradation [21]. However, incorporating water-binding components such as hydrocolloids or sugars effectively reduces syneresis in starch-based gels [29,30,31].

Research has demonstrated gellan gum’s effectiveness in reducing gel syneresis compared to agar and gelatin alternatives [4]. The high-acyl gellan gum utilized in this investigation consists of water-soluble polymers containing O-2-glyceryl and O-5-acetyl groups on (1→3)-linked glucose residues, which effectively prevent compact double helix formation and polymer chain association [31]. Consequently, high-acyl gellan gum serves as a valuable ingredient, independently or in combination with other gelling agents, for syneresis reduction and texture enhancement in food applications. Studies have confirmed gellan gum’s synergistic effectiveness with sweet potato starch in minimizing syneresis compared to κ-carrageenan [32]. Different gums exhibited varying syneresis patterns in milk pudding formulations (Figure 2). κ-carrageenan initially presents as irregular coils in heated solutions, forming three-dimensional matrices through helical junction zones upon cooling [33]. Continued cooling promotes junction zone aggregation, resulting in rigid gel formation. These molecular associations may contribute to increased syneresis and reduced stability in modified corn starch gels.

In general, research indicates that increasing starch concentration correlates with decreased gel strength (*p* < 0.05), breaking force (*p* < 0.05), and pudding rigidity. This phenomenon might result from elevated levels of highly branched amylopectin structures in modified waxy corn starch. The increased amylopectin content inhibits hydrocolloid interactions, limiting gelatinized starch–polysaccharide associations (Table 3). These findings align with published research examining the effect of starch on carrageenan gel distortion behavior [34]. De Vries [35] documented a reduced break strength in κ-carrageenan (0.2%) milk pudding gels containing 2% starch additions, attributing this to gelation interference. Lai et al. [36] reported diminished gel strength with increasing rice starch incorporation in 2% κ-carrageenan gels, citing weak gel formation between starch and hydrocolloid networks. Starch traditionally serves as a thickening agent in heated food processing, enhancing water retention, gel syneresis, freeze–thaw stability, and textural properties. The modified waxy corn starch utilized in this study exhibits retrogradation tendencies during refrigerated storage, particularly regarding amylose behavior. The addition of modified waxy corn starch to various gums consistently resulted in significant reductions (*p* < 0.05) in gel penetration parameters (Table 3). The penetration characteristics of modified waxy corn starch and gum (0.3%) combinations may be influenced by additional factors, including cream, eggs, cationic concentrations, granular disruption extent, and gum proportions in complex food gel systems, warranting further investigation.

The observed increase in % syneresis may be attributed to the molecular rearrangement of modified waxy corn starch through hydrogen bonding between amylopectin chains [37]. κ-carrageenan and ι-carrageenan represent the most widely utilized carrageenan variants in food applications. ι-carrageenan, characterized by higher sulfation at the C2 position of 1,4-linked 3,6-anhydro-α-D-galactopyranose, produces soft weak gels, while κ-carrageenan forms robust brittle structures [38]. These molecular sulfation differences influence syneresis behavior and gel strength characteristics (Figure 2 and Table 2). ι-carrageenan demonstrated superior syneresis resistance and water retention compared to κ-carrageenan in 5% and 7.5% modified waxy corn starch systems during extended refrigerated storage (Figure 3). Gellan gum and ι-carrageenan formulations exhibited minimal syneresis increases with elevated modified waxy corn starch concentrations throughout the storage period. The high-acyl gellan gum employed produced soft weak gel structures while effectively minimizing syneresis in formulations containing elevated modified waxy corn starch levels (7.5%) during refrigeration (Figure 3). The enhanced syneresis resistance of ι-carrageenan and gellan gum formulations can be attributed to their respective high sulfation degrees and acyl group content, facilitating extensive hydrogen bonding with water molecules. Despite utilizing modified waxy corn starch, amylopectin demonstrated retrogradation tendencies during refrigerated storage, with higher concentrations promoting increased syneresis.

It is shown that commercial milk pudding formulations MA and MB demonstrated superior syneresis resistance compared to experimental hydrocolloid combinations during 7-day storage (Figure 1, Figure 2, and Figure 3a). However, 0.3% ι-carrageenan and 0.3% gellan gum formulations containing 1% modified corn starch exhibited enhanced syneresis resistance compared to commercial products during 14-day storage (Figure Figure 1, Figure 2, and Figure 3b). The syneresis measurements in this study consistently exceeded 1%, indicating higher levels compared to binary hydrocolloid gel systems (0.5 wt%) comprising agar, gellan gum, and gelatin stored at 4 °C for 7 and 14 days, which exhibited values below 1% [4]. These findings suggest the potential for developing an optimized binary hydrocolloid milk pudding formulation with an increased hydrocolloid gel concentration of 0.5%.

Sucrose demonstrated minimal effects on penetration parameters in ι-carrageenan and gelatin formulations, though it generally enhanced the overall penetration characteristics. The precise mechanism of sucrose influence on starch gel networks, whether through direct interchain effects or indirect amylose leaching modulation during gelatinization, remains unclear [39]. Incorporating 5–10% sucrose increased κ-carrageenan konjac (7:3) milk pudding firmness while promoting denser network formation [40]. Sucrose significantly influences gel structure and texture in confectionery and dessert applications, enhancing gellan gel network strength and complementing the calcium ion stabilization of ordered network structures [41]. Maximum rupture force increased with 0–60% sucrose addition in 0.5% carrageenan gels. However, sharp decreases were observed in kappa-carrageenan locust bean gum systems at 60% sucrose, potentially due to water competition between sucrose and gum molecules [42].

Our results demonstrate that commercial milk puddings exhibited higher breaking points (8.75–9.82 mm, Table 1) compared to experimental formulations (4.85–7.27 mm, Table 2, Table 3 and Table 4). Breaking point measurements represent the distance between the initial probe contact and breaking force determination. Commercial products demonstrated superior deformation capacity compared to experimental formulations containing various hydrocolloid and sucrose combinations. While penetration testing provides empirical data rather than fundamental mechanical properties [43], the combined analysis of penetration rigidity, breaking force, and breaking point offers valuable qualitative insights into formulation effects on pudding characteristics. Higher breaking force and gel strength seem to correlate with increased syneresis, particularly in agar and κ-carrageenan systems. Penetration measurements demonstrate superior correlation with sensory texture compared to small deformation fundamental testing [43]. Future research opportunities include investigating interactions between gum combinations (gelatin/agar, gelatin/gellan, agar/gellan) at 0.5% concentrations and gum–starch interactions (modified corn starch with gellan, agar, or gelatin) at 0.5% levels regarding penetration characteristics and syneresis behavior. Previous research has established agar’s predominant influence on gel strength, breaking force, and rigidity among binary hydrocolloid systems, while gellan gum demonstrated superior syneresis resistance during 7- and 14-day storage [4]. Binary hydrocolloid systems exhibited significant breaking point increases with 0–0.5% gellan gum addition. These findings provide valuable insights for optimizing gum and modified corn starch combinations in refrigerated pudding development, particularly regarding gel strength, rigidity, breaking force, breaking point, texture profiles, and syneresis characteristics.

### 4.2. Sensory Evaluation of Milk Pudding

An analysis of sugar content revealed significant impacts on the sensory properties of jelly dessert, including flavor, texture, and overall acceptability, presenting challenges in maintaining consistent product attributes [44]. Based on these findings, a standardized sucrose content of 5% was established for the initial sensory evaluation of milk pudding with various polysaccharide compositions.

Research by Lethuaut et al. [14] demonstrated that variations in carrageenan composition influenced sweetness perception at elevated sucrose concentrations (10%), with λ-carrageenan desserts exhibiting the highest sweetness intensity and ι-carrageenan desserts the lowest. In the comprehensive sensory evaluation, milk pudding formulated with gellan gum achieved superior ratings across multiple parameters, including color, springiness, and overall acceptability, compared to other polysaccharide variants.

The sensory evaluation revealed that sample B75 received lower sweetness preference scores compared to B50. This indicates a consumer preference for milk pudding containing 5% sucrose over formulations with 2.5% or 7.5% sucrose content, suggesting that current commercial products may exceed optimal sweetness levels. The findings also emphasize the importance of achieving balanced mechanical properties, specifically in terms of breaking force and gel strength, while maintaining high springiness and overall acceptability scores. A comparative analysis showed no statistically significant differences in sweetness preference between commercial milk pudding MA and the experimental formulation B25 (2.5% sucrose, 0.3% gellan gum, 1% modified corn starch). Using a nine-point hedonic scale, the 5% sucrose formulation received scores ranging from 5.23 to 5.70, indicating moderate consumer acceptance. However, the commercial product (MA) maintained superior ratings across all sensory attributes among the four variants evaluated (Table 6).

## 5. Conclusions

The research findings demonstrate the beneficial synergistic interactions between agar, κ-carrageenan, modified corn starch, and sucrose in enhancing the structural properties of milk pudding, specifically regarding breaking force, rigidity, and gel strength; however, this improved interaction correlates with increased syneresis in the final product. Storage studies indicate that milk pudding formulated with 0.3% gellan gum and maintained at 4 °C for 7–14 days exhibits reduced syneresis while maintaining acceptable gel strength and rigidity parameters, ultimately improving overall consumer acceptance. The observed decrease in breaking force and gel strength following 7-day refrigerated storage in samples with increased modified corn starch content (1–5%) may be attributed to molecular reorganization within the starch component. The experimental data support the role of sucrose in enhancing the structural properties of milk pudding containing κ-carrageenan, gellan gum, and agar. Additional research is warranted to fully characterize the starch–hydrocolloid interactions and their influence on texture development and syneresis control.

## Figures and Tables

**Table 1:** Textural characteristics ^1^ of 3 commercial milk pudding penetration tests.

Commercial Milk Pudding	Breaking Force (B.F.)	Breaking Point (B.P.)	Gel Strength (G.S.)	Rigidity (R.)
	(g)	(mm)	(g × mm)	(g/mm)
MA	15.0 ± 0.5 ^b^	8.75 ± 0.18 ^a^	131 ± 6 ^b^	1.72 ± 0.03 ^b^
MB	10.7 ± 0.4 ^a^	8.76 ± 0.25 ^a^	94 ± 5 ^a^	1.23 ± 0.04 ^a^
MC	17.4 ± 0.3 ^c^	9.82 ± 0.36 ^b^	171 ± 9 ^c^	1.77 ± 0.04 ^c^

^1^ Gel strength (G.S.) = (Breaking force × Breaking point); Rigidity = (Breaking force/Breaking point). Data are expressed as mean (n = 6). Different superscripts in the same column indicate significant differences.

**Table 3:** Variation in penetration test parameters ^1^ of milk pudding as a function of different polysaccharide and modified corn starch concentrations (mean ± SD, n = 6).

Type of Polysaccharide	Modified Corn Starch	Breaking Force (B.F.)	Breaking Point (B.P.)	Gel Strength (G.S.)	Rigidity (R.)
	(%)	(g)	(mm)	(g × mm)	(g/mm)
κ-carrageenan	1.0	22.7 ± 0.3 ^c^	5.41 ± 0.14 ^c^	123 ± 4 ^c^	4.20 ± 0.10 ^b^
	3.0	21.4 ± 0.8 ^b^	5.12 ± 0.13 ^b^	109 ± 3 ^b^	4.18 ± 0.24 ^b^
	5.0	17.9 ± 0.6 ^a^	4.85 ± 0.04 ^a^	86 ± 2 ^a^	3.69 ± 0.12 ^a^
ι-carrageenan	1.0	11.7 ± 0.3 ^c^	6.34 ± 0.13 ^a^	74 ± 3 ^c^	1.84 ± 0.02 ^c^
	3.0	10.5 ± 0.4 ^b^	6.27 ± 0.22 ^a^	65 ± 3 ^b^	1.68 ± 0.10 ^b^
	5.0	8.38 ± 0.4 ^a^	7.27 ± 0.25 ^b^	60 ± 2 ^a^	1.16 ± 0.07 ^a^
Gellan gum	1.0	18.8 ± 0.5 ^c^	5.23 ± 0.10 ^a^	98 ± 3 ^c^	3.59 ± 0.12 ^c^
	3.0	16.1 ± 0.4 ^b^	5.48 ± 0.17 ^b^	88 ± 3 ^b^	2.94 ± 0.11 ^b^
	5.0	13.6 ± 0.6 ^a^	5.43 ± 0.07 ^b^	73 ± 3 ^a^	2.51 ± 0.09 ^a^
Gelatin	1.0	14.6 ± 0.5 ^c^	6.25 ± 0.14 ^b^	91 ± 4 ^c^	2.34 ± 0.08 ^a^
	3.0	13.5 ± 0.5 ^b^	5.37 ± 0.34 ^a^	72 ± 4 ^b^	2.53 ± 0.21 ^a^
	5.0	12.5 ± 0.4 ^a^	5.25 ± 0.19 ^a^	65 ± 2 ^a^	2.38 ± 0.13 ^a^
Agar	1.0	26.6 ± 0.4 ^c^	5.37 ± 0.02 ^a^	143 ± 2 ^c^	4.95 ± 0.06 ^c^
	3.0	24.6 ± 0.5 ^b^	5.49 ± 0.12 ^ab^	135 ± 5 ^b^	4.48 ± 0.08 ^b^
	5.0	20.4 ± 0.5 ^a^	5.55 ± 0.16 ^b^	113 ± 3 ^a^	3.68 ± 0.15 ^a^

^1^ Gel strength (G.S.) = (Breaking force × Breaking point); Rigidity = (Breaking force / Breaking point). Data are expressed as mean (n = 6). Different letters in the same column indicate significant differences.

**Table 4:** Variation in penetration test parameters ^1^ of milk pudding as a function of polysaccharide and different sucrose concentrations (mean ± SD, n = 6).

Type of Polysaccharide	Sucrose	Breaking Force (B.F.)	Breaking Point (B.P.)	Gel Strength (G.S.)	Rigidity (R.)
	(%)	(g)	(mm)	(g × mm)	(g/mm)
κ-arrageenan	2.5	20.4 ± 0.8 ^a^	5.03 ± 0.15 ^a^	103 ± 4 ^a^	4.05 ± 0.24 ^a^
	5.0	21.4 ± 0.8 ^b^	5.12 ± 0.13 ^a^	109 ± 3 ^b^	4.18 ± 0.24 ^a^
	7.5	22.6 ± 0.4 ^c^	5.32 ± 0.15 ^b^	120 ± 5 ^c^	4.25 ± 0.08 ^a^
ι-carrageenan	2.5	9.83 ± 0.7 ^a^	6.17 ± 0.16 ^a^	60.6 ± 4 ^a^	1.60 ± 0.14 ^a^
	5.0	10.5 ± 0.4 ^a^	6.27 ± 0.22 ^a^	65.9 ± 3 ^b^	1.68 ± 0.10 ^a^
	7.5	11.1 ± 0.5 ^a^	6.30 ± 0.18 ^a^	70.2 ± 3 ^b^	1.77 ± 0.08 ^a^
Gellan gum	2.5	15.5 ± 0.4 ^a^	5.38 ± 0.12 ^a^	83.4 ± 2 ^a^	2.88 ± 0.11 ^a^
	5.0	16.1 ± 0.4 ^b^	5.48 ± 0.17 ^ab^	88.2 ± 3 ^b^	2.94 ± 0.11 ^a^
	7.5	16.8 ± 0.2 ^c^	5.62 ± 0.12 ^b^	94.4 ± 2 ^c^	2.29 ± 0.07 ^a^
Gelatin	2.5	13.1 ± 0.8 ^a^	5.16 ± 0.13 ^a^	67.5 ± 3 ^a^	2.54 ± 0.14 ^a^
	5.0	13.5 ± 0.5 ^ab^	5.37 ± 0.34 ^a^	72.6 ± 4 ^b^	2.53 ± 0.21 ^a^
	7.5	14.0 ± 0.6 ^b^	5.41 ± 0.16 ^a^	75.9 ± 4 ^b^	2.60 ± 0.12 ^a^
Agar	2.5	22.8 ± 1.2 ^a^	5.27 ± 0.18 ^a^	120 ± 4 ^a^	4.34 ± 0.35 ^a^
	5.0	24.6 ± 0.5 ^b^	5.49 ± 0.12 ^b^	135 ± 5 ^b^	4.48 ± 0.08 ^ab^
	7.5	27.3 ± 1.0 ^c^	5.75 ± 0.10 ^c^	157 ± 4 ^c^	4.76 ± 0.25 ^b^

^1^ Gel strength (G.S.) = (Breaking force × Breaking point); Rigidity = (Breaking force/Breaking point). Data are expressed as mean (n = 6). Different superscripts in the same column indicate significant differences.

**Table 5:** Sensory evaluation ^1^ of commercial milk pudding products and different polysaccharides of milk pudding stored at 4 °C for 7 days.

Milk Pudding	Color	Flavor	Sweetness	Springiness	Overall Acceptability
Commercial MA	6.90 ± 0.99 ^a^	6.83 ± 1.15 ^a^	5.40 ± 1.07 ^a^	6.60 ± 1.19 ^a^	7.00 ± 1.01 ^a^
Commercial MB	5.77 ± 1.19 ^b^	5.97 ± 1.22 ^b^	5.17 ± 1.26 ^a^	5.53 ± 1.14 ^b^	5.73 ± 0.98 ^b^
Commercial MC	5.47 ± 1.31 ^b^	5.47 ± 1.41 ^b^	5.13 ± 1.22 ^a^	5.10 ± 1.06 ^b^	5.23 ± 1.19 ^b^
κ-carrageenan	5.10 ± 1.15 ^c^	5.67 ± 1.24 ^b^	5.25 ± 1.38 ^a^	4.20 ± 1.19 ^b^	4.53 ± 1.09 ^c^
ι-carrageenan	5.64 ± 0.89 ^b^	5.83 ± 1.14 ^b^	5.39 ± 1.11 ^a^	5.22 ± 1.05 ^b^	5.25 ± 1.14 ^b^
Gellan gum	5.75 ± 0.62 ^b^	5.51 ± 1.36 ^b^	5.41 ± 1.18 ^a^	5.55 ± 0.89 ^b^	5.72 ± 1.00 ^b^
Gelatin	5.27 ± 1.19 ^c^	5.47 ± 1.32 ^b^	5.16 ± 1.36 ^a^	5.31 ± 1.17 ^b^	5.13 ± 0.92 ^b^
Agar	5.12 ± 1.14 ^c^	5.58 ± 1.11 ^b^	4.87 ± 1.22 ^a^	4.07 ± 1.29 ^b^	4.22 ± 1.28 ^c^

^1^ A 9-point hedonic scale. 1 = dislike extremely; 9 = like extremely. Each polysaccharide milk pudding contains 1% modified corn starch and 5% sucrose. Data are expressed as mean (n = 30). Different superscripts in the same column indicate significant differences.

**Table 6:** Sensory evaluation of milk pudding made by different concentrations of sucrose stored at 4 °C for 7 days.

Type of Milk Pudding	Color	Flavor	Sweetness	Springiness	Overall Acceptability
Commercial milk pudding (MA)	6.17 ± 1.18 ^b^	5.77 ± 1.25 ^b^	5.17 ± 1.49 ^ab^	6.07 ± 1.36 ^b^	7.03 ± 1.27 ^b^
Milk pudding (B25) with 2.5% sucrose, 0.3% gellan, and 1% modified corn starch	5.30 ± 1.64 ^a^	4.87 ± 1.59 ^a^	5.07 ± 1.44 ^ab^	5.17 ± 1.26 ^a^	5.07 ± 1.28 ^a^
Milk pudding (B50) with 5.0% sucrose, 0.3% gellan, and 1% modified corn starch	5.23 ± 1.65 ^a^	5.37 ± 1.56 ^ab^	5.70 ± 1.12 ^b^	5.73 ± 1.51 ^ab^	5.63 ± 1.22 ^a^
Milk pudding (B75) with 7.5% sucrose, 0.3% gellan, and 1% modified corn starch	5.47 ± 1.57 ^ab^	5.43 ± 1.43 ^ab^	4.80 ± 1.58 ^a^	5.40 ± 1.35 ^ab^	5.40 ± 1.45 ^a^

A 9-point hedonic scale. 1 = dislike extremely; 9 = like extremely. Data are expressed as mean (n = 30). Different superscripts in the same column indicate significant differences.

## Data Availability

The data presented in this work are available upon request from the corresponding author.

## References

[1] de Wijk R.A., Van Gemert L.J., Terpstra M.E.J., Wilkinson C.L. (2003). Texture of semi-solids; sensory and instrumental measurements on vanilla custard desserts. Food Qual. Prefer..

[2] Sheidae Z., Sarmadi B., Hosseini S.M., Javanmardi F., Kianoush K.D., Mortazavian A.M. (2020). Influence of κ-carrageenan, modified starch and inulin addition on rheological and sensory properties of non-fat and non-added sugar dairy dessert. Curr. Nutr. Food Sci..

[3] Imeson A.P., Philips G.O., Williams P.A. (2000). Carrageenan. Handbook of Hydrocolloids.

[4] Lin H.T., Tsai J.S., Liao H.H., Sung W.C. (2023). The effect of hydrocolloids on penetration tests and syneresis of binary gum gels and modified corn starch-gum gels. Gels.

[5] Langendorff V., Cuvelier G., Michon C., Launay B., Parker A., De Kruif C.G. (2017). Effects of carrageenan type on the behaviour of carrageenan/milk mixtures. Food Hydrocoll..

[6] Verbeken D., Bael K., Thas O., Dewettinck K. (2006). Interactuions between κ-carrageenan, milk proteins and modified starch in sterilized dairy desserts. Int. Dairy J..

[7] Agoda-Tandjawa G., Le Garnec C., Boulenguer P., Gilles M., Langendorff V. (2017). Rheological behavior of starch/carrageenan/milk proteins mixed systems: Role of each biopolymer type and chemical characteristics. Food Hydrocoll..

[8] Depypere F., Verbeken D., Thas O., Dewettinck K. (2003). Mixture design approach on the dynamic rheological and uniaxial compression behaviour of milk desserts. Food Hydrocoll..

[9] Verbeken D., Thas O., Dewettinck K. (2004). Textural properties of gelled dairy desserts containing κ-carrageen and starch. Food Hydrocoll..

[10] Mahmood K., Kamilah H., Shang P.L., Sulaiman S., Ariffin F., Alias A.K. (2017). A review: Interaction of starch/non-starch hydrocolloid blending and the recent food applications. Food Biosci..

[11] Sodini I., Remeuf F., Haddad S., Corrieu G. (2004). The relative effect of milk base, starter, and process on yogurt texture: A review. Crit. Rev. Food Sci. Nutr..

[12] Marta H., Cahyana Y., Djali M. (2020). The effect of starch-hydrocolloid interaction on starch digestibility, pasting and physicochemical properties: A review. IOP Conf. Ser. Earth Environ. Sci..

[13] Christianson D.D., Lineback R.D., Inglett G.E. (1982). Hydrocolloid interactions with starches. Food Carbohydrates.

[14] Lethuant L., Brossard C., Rousseau F., Bousseau B., Genot C. (2003). Sweetness-texture interactions in model dairy desserts: Effect of sucrose concentration and the carrageenan type. Int. Dairy J..

[15] Nguyen P.T.M., Kravchuk O., Bhandari B., Prakash S. (2017). Effect of different hydrocolloids on texture, rheology, tribology and sensory perception of texture and mouthfeel of low-fat pot-set yoghurt. Food Hydrycoll..

[16] Supavititpatana P., Wirjantoro T.I., Apichartsrangkoon A., Raviyan P. (2008). Addition of gelatin enhanced gelation of corn-milk yogurt. Food Chem..

[17] Duboc P., Mollet B. (2001). Application of exopolysaccharides in the dairy industry. Int. Dairy J..

[18] Bagal-Kestwal D.R., Pan M.H., Chiang B.H., Visakh P.M., Bayraktar O., Menon G. (2019). Properties and applications of gelatin, Pectin, and carrageenan gels. Bio Monomers for Green Polymeric Composite Materials.

[19] Therkelsen G.H., Whister R.L., BeMiller J.N. (1993). Carrageenan. Industrial Gums.

[20] Richardson R.K., Goycoolea F.M. (1994). Rheological measurement of κ-carrageenan during gelation. Carbohydr. Polym..

[21] Chen H.H., Xu S.Y., Wang Z. (2006). Gelation properties of flaxseed gum. J. Food Eng..

[22] Karim A.A., Norziah M.H., Seow C.C. (2000). Methods for the study of starch retrogradation. Food Chem..

[23] Tang Q., Roos Y.H., Miao S. (2024). Comparative studies of structural and thermal gelation behaviours of soy, lentil and whey protein: A pH-dependency evaluation. Food Hydrocoll..

[24] Charoenrein S., Tatirat O., Muadklay J. (2008). Use of centrifugation-filtration fordetermination of syneresis in freeze-thaw starch gels. Carbohydr. Polym..

[25] Moskowitz H.R. (1982). Sensory intensity versus hedonic functions: Classical psychophysical approaches. J. Food Qual..

[26] Junaid M., Javed I., Abdullah M., Gulzar M., Younas U., Nasir J., Ahmad N. (2013). Development and quality assessment of flavored probiotic acidophilus milk. J. Anim. Plant Sci..

[27] Morris V.J. (1990). Starch gelation and retrogradation. Trends Food Sci. Technol..

[28] Xu X., Ye S., Zuo X., Fang S. (2022). Impact of guar gum and locust bean gum addition on the pasting, rheological properties, and freeze-thaw stability of rice starch gel. Foods.

[29] Arunyanart T., Charoenrein S. (2008). Effect of sucrose on the freeze-thaw stability of rice starch gels: Correlation with microstructure and freezeable water. Carbohydr. Polym..

[30] Baker L.A., Rayas-Duarte P. (1998). Freeze-thaw stability of amaranth starch and the effects of salt and sugars. Cereal Chem..

[31] Zhang C., Lim S.T., Chung H.J. (2019). Physical modification of potato starch using mild heating and freezing with minor addition of gums. Food Hydrocoll..

[32] Mao R., Tang J., Swanson B.G. (2000). Texture properties of high and low acyl mixed gellan gels. Carbohydr. Polym..

[33] Lee M.H., Baek M.H., Cha D.S., Park H.J., Lim S.T. (2002). Freeze-thaw stabilization of sweet potato starch gel by polysaccharide gums. Food Hydrocoll..

[34] Dogsa I., Cerar J., Jamnik A., Tomsic M. (2017). Supramolecular structure of methyl cellulose and lambda- and kappa- carrageenan in water: SAXS study using the string-of-beads model. Carbohydr. Polym..

[35] de Vries J.A. (2002). Interactions of starch and other hydrocolloids. Carbohydr. Neth..

[36] Lai V.M.F., Hung A.L., Lii C.Y. (1999). Rheological properties and phase transition of red algal polysaccharide starch composites. Food Hydrocoll..

[37] He H., Zhang Y., Hong Y., Gu Z. (2015). Effects of hydrocolloids on corn starch retrogradation. Starch/Starke.

[38] Geonzon L.C., Kobayashi M., Tassieri M., Bacabac R.G., Adachi Y., Matsukawa S. (2023). Microrheological properties and local structure of ι-carrageenan gels probed by using optical tweezers. Food Hydrocoll..

[39] Prokopowich D.J., Biliaderis C.G. (1995). A comparative study of the effect of sugars on the thermal and mechanical properties of concentrated waxy maize, wheat, potato and pea starch gels. Food Chem..

[40] Wang X., Zhou D., Guo Q., Liu C. (2021). Textural and structure properties of a κ-carrageenan-konjac gum mix gel: Effects of κ-carrageenan concentration, mixing ratio, sucrose and Ca^2+^ concentrations and its application in milk pudding. J. Sci. Food Agric..

[41] Bayarri S., Costell E., Duran L. (2002). Influence of low sucrose concentrations on the compression resistance of gellan gum gels. Food Hydrocoll..

[42] Fiszman S.M., Duran L. (1989). Mechanical properties of kappa carrageen-locust bean gum mixed gels with added sucrose. Food Hydrocoll..

[43] Nishinari K., Fang Y., Rosenthal A. (2019). Human oral processing and texture profile analysis parameters: Bridging the gap between the sensory evaluation and the instrumental measurements. J. Texture Stud..

[44] Ekpong A., Ngarmsak T., Winger R.J. (2006). Comparing sensory methods for the optimization of mango gel snacks. Food Qual. Prefer..

